# The relationship between childhood traumatic experience and suicidal tendency in non-suicidal self-injury behavior patients

**DOI:** 10.1186/s12888-023-04863-0

**Published:** 2023-06-05

**Authors:** Fang Cheng, Linwei Shi, Shujun Wang, Qiong Jin, Huabing Xie, Beini Wang, Wenwu Zhang

**Affiliations:** 1 Department of Pediatric Psychology, Ningbo Kangning Hospital, Ningbo, 315201 Zhejiang China; 2 School of Mechanical Engineering and Automation, Fuzhou University, Fuzhou, 350116 China; 3 Department of General Medicine, People’s Hospital of Wuhan University, Wuhan, 430060 Hubei China

**Keywords:** Suicidal tendency, Childhood traumatic experience, Psychiatric comorbidity mechanism, NSSI, Structural equation model

## Abstract

**Background:**

Individuals with non-suicidal self-injury (NSSI) behavior are usually prone to repeated, intentional, direct harm to their own bodies that is not allowed by society without suicidal ideation. Under this behavior guidance, childhood traumatic experience may easily cause a series of psychological comorbidity symptoms, such as anxiety and depression, finally leading to a suicidal tendency.

**Methods:**

A total of 311 adolescent NSSI behavioral patients were recruited at the Ningbo Kangning hospital, Zhejiang Province according to the DSM-5 diagnostic criteria. Demographic data, childhood abuse and neglect, internet addiction, self-esteem, anxiety, and suicidal tendency were evaluated. A structural equation model with a path induction mechanism was constructed to evaluate the relationship between distal and proximal factors related to suicidal tendencies due to childhood traumatic experiences in NSSI behavioral individuals.

**Results:**

Among the 311 subjects included in the survey, 250 (80.39%) suffered traumatic experiences, such as emotional abuse/physical abuse/sexual abuse/emotional neglect or physical neglect in their childhood, 303 (97.43%) had suicidal ideation, 271 (87.14%) showed the total score of self-esteem, 148 (47.59%) had different degrees of Internet addiction tendency, and 286 (91.96%) showed obvious anxiety. The established path model fit well (GFI = 0.996, RMSEA = 0.03), and the model showed that self-esteem, anxiety, and childhood traumatic experience had standardized coefficients of -0.235 (z = -4.742, *p* < 0.01), 0.322 (z = 6.296, *p* < 0.01), 0.205 (z = 4.047, *p* < 0.01), respectively, with suicidal ideation path, suggesting that self-esteem, Internet addiction, and anxiety showed significant mediating effects in the process of childhood traumatic experience affecting suicidal ideation.

**Conclusion:**

In the context of childhood traumatic experience, it is often accompanied by a series of regulatory behaviors such as Internet addiction, self-esteem, and so on, which finally leads to anxiety, mental symptoms, and even suicidal tendencies. The results provide effective support for the structural equation modeling to evaluate the multi-level influence of NSSI behavior individuals and emphasize that childhood familial factors may lead to psychiatric comorbidity symptoms and suicidal behavior.

## Introduction

Non-suicidal self-injury (NSSI) is the intentional, direct injury to body tissues without suicidal intent—an act that is socially and culturally unacceptable. Cutting is the most predominant form of NSSI, and other injury types include burns, scrapes or scratches to the skin, and bites [1]. Although most NSSI behaviors do not result in the immediate death of the affected individual, it is associated with multiple clinical dysfunctions that cause significant suffering to family and friends [2]. Also, NSSI is a significant predictor of subsequent suicidal behavior [3, 4], with the risk of suicide in the first year after self-injury being 66 times the annual risk in the general population [5–8] and the risk of suicide 5, 10, and 15 years after self-harm being 1.7%, 2.4%, and 3.0%, respectively [9]. Therefore, it is necessary to investigate the etiology and potential risk factors for the development of suicidal ideation in adolescents diagnosed with NSSI.

Suicidal ideation involves genetic, biological, spiritual, psychological, physical, social, and cultural factors, and they lead to suicidal ideation through complex interactive processes [10, 11]. As a common and important risk factor for patients with suicidal tendencies, childhood trauma is one of the predominant topics of research. Childhood trauma is an adverse early life experience that can affect the victim's psychological state via the behavioral, emotional, and cognitive pathways, increasing the risk of developing mental illness in adulthood [12]. High levels of childhood trauma, in addition to being associated with the occurrence of NSSI [13, 14], are strongly associated with subsequent suicidal behaviors and styles [15]. Childhood trauma includes multiple forms like emotional abuse, physical abuse, sexual abuse, physical neglect, and emotional neglect. The types of childhood trauma may not exist singularly in an individual, and they all play a role in the etiology of NSSI. A general population survey in Germany showed that about 3% of the individuals had a history of self-injurious behavior at least once during their lifetime, and about 65% of them had experienced at least one type of childhood trauma, with emotional abuse being the predominant type, and about 50% of the individuals experienced multiple types of childhood trauma [16]. A longitudinal study showed that young children who experienced physical neglect and maltreatment had a higher risk of suicide [17]. Another study confirmed the association between sexual abuse and NSSI and attempted early intervention to reduce the occurrence of suicide [18]. A study in Turkey covering 651 secondary school students showed that a history of childhood neglect increased the risk of NSSI and physical and sexual abuse increased the risk of suicide [19]. A cross-sectional study of secondary school students in mainland China showed that high fatal self-injury was associated with childhood sexual abuse, emotional abuse, and emotional neglect, and low fatal self-injury was associated with childhood sexual abuse [20].

The specific psychological mechanisms underlying the relationship between childhood trauma and suicidal ideation are currently unknown. However, it has been established that the relationship is mediated in part by comorbidities [21], specifically, childhood traumatic experiences may lead to mood disorders such as depression, anxiety, and personality disorders, which in turn lead to the development of adolescent psychopathology. Even Internet addiction is linked to the development of suicidal ideation as a moderator of childhood traumatic experiences [22]. Paul S. Links et al. believe that when patients with borderline personality disorder (BPD) have different degrees of suicidal ideation and behavior, they are often accompanied by a series of unstable emotional factors such as depression, anxiety, and low self-esteem [23]. In a recent study in China, it was found that childhood trauma has both direct and indirect effects on suicidal ideation, with Internet addiction playing a mediating role in these effects. Similarly, another Chinese study demonstrated the mediating role of anxiety [24]. The emotion regulation model of suicidal ideation also elucidates the mediating role of anxiety [25], and the interpersonal stress caused by Internet addiction may increase the severity of emotional distress (e.g., anxiety) and emotion regulation deficits, thereby increasing the risk of suicide. Research has also shown that low self-esteem is a common characteristic of individuals with self-injurious behaviors or a history of self-injury and that individuals with self-injurious behaviors have lower self-esteem compared to individuals without self-injurious behaviors [26]. Negative self-expression, peer/parental rejection, alienation, and declining self-esteem may be responsible for self-injurious behaviors in some young people [27], and childhood abuse may weaken self-esteem, self-concept, interpersonal relationships, trust capacity, and make it easier to develop disappointment, shame, and self-loathing, which can lead to NSSI by making them believe that they deserve to be punished and even suicidal behavior. Although research has linked anxiety, Internet addiction, and self-esteem to childhood trauma and NSSI as well as suicide, it is unclear how childhood trauma relates to suicidal ideation in the context of NSSI, making it necessary to explore the interactions between them.

In the current work, we constructed a structural equation model with pathway induction mechanisms aimed to assess the relationship between distal and proximal factors of suicidality in the presence of traumatic childhood experiences (TCE) in individuals with NSSI behaviors. A total of 311 patients with NSSI behavior were selected and demography, childhood abuse and neglect, internet addiction, self-esteem psychology, anxiety, and suicidal tendency were assessed. The purpose of this study was to develop a conceptual model of childhood traumatic experiences and risk factors for suicidal ideation to help clinicians better understand the lifelong trajectory of self-harm behaviors and to develop and implement more targeted interventions. Also, this study may contribute to the development of a model of childhood traumatic experiences and the etiology of NSSI.

## Methods

### Ethics statement

Written informed consent was obtained from all participants and their parents before initiating the study, including specific mention of the potential triggering nature of all relevant issues, and confidentiality restrictions related to mandatory reporting of concerns. The study protocol was approved by the Ethics Committee of the Ningbo Kangning Hospital (approval number: NBKNYY-2021-LC-32).

### Subjects

The subjects were adolescents with NSSI (no history of outpatient visits) who presented to the outpatient clinic of the Kangning Hospital in Ningbo, Zhejiang Province, China between September 2020 and December 2021. The subjects were sequentially enrolled and diagnosed by Dr. Fang Cheng, the deputy chief psychiatrist, and Dr. Wenwu Zhang, the chief psychiatrist, and the purpose and significance of the study were explained to them in detail before the assessments were administered by Dr. Wenwu Zhang, the attending psychiatrist.

The inclusion criteria were as follows: (1) patients who met the DSM-5 diagnostic criteria for NSSI; (2) first-time patients who had not received any form of treatment for NSSI behavior; (3) patients aged 11–16 years; (4) whose parents/ guardians provided informed consent.

The exclusion criteria were as follows: (1) severe physical or organic brain diseases; (2) comorbid psychiatric disorders other than anxiety, depressive disorders, and Internet addiction; (3) history of severe substance abuse.

### Survey content

In total, 311 adolescents (average age 14.49 ± 1.51 years) with varying degrees of NSSI behaviors were included in the study. In the current study, TCE (abuse and neglect), suicidal tendencies, the degree of self-esteem, Internet addiction, and anxiety were analyzed.

#### Childhood trauma questionnaire short form

Childhood traumatic experiences often refer to abuse (physical) and neglect (psychological) during childhood, including emotional abuse, physical abuse, sexual abuse, and emotional and physical neglect, which have profound effects on physical and mental health even in adulthood. The Childhood Trauma Questionnaire Short Form (CTQ-SF) [28] was chosen to assess subjects' experiences of childhood abuse and neglect. The form consists of 28 items, each rated on a five-point scale: 1, never; 2, occasionally; 3, sometimes; 4, often; 5, always. It covers five subscales of emotional abuse, physical abuse, sexual abuse, emotional neglect, and somatic neglect. With emotional abuse scores ≥ 13, physical abuse scores ≥ 10, sexual abuse scores ≥ 8, the Cronbach's alpha coefficient for the CTQ-SF was 0.791, and the Cronbach's alpha coefficients for the five subscales ranged from 0.403 to 0.697.

#### Beck suicidal ideation scale

Suicidal ideation is the presence of death, suicide, and severe self-harm in thoughts or intentions, including thoughts about the plans, steps, and outcomes of suicidal behavior [28]. In the current study, the Beck Suicidal Ideation Scale was used to assess suicidal ideation. The scale consists of 38 items with entries on a 3-point scale and a 4-point scale that assess the severity of suicidal ideation and risk of suicide alone (on a scale of 0–100) in the most recent week and at the height of the depression. The subjects responded to the first 5 items, and if the response to both items 4 and 5 was "no", then the subject was considered to have no suicidal ideation. If the response to either item 4 or 5 was "weak" or "moderate to strong", then the subject was considered to have suicidal ideation and needed to respond to the next 14 items. The intensity of suicidal ideation is based on the mean response to items 1 to 5, with higher scores indicating a greater intensity of suicidal ideation. The risk of suicide was assessed using responses to items 6 to 19, and the higher the score, the greater the risk of suicide. Suicidal ideation severity in the most recent week α = 0.63 (*n* = 1801), ICC = 0.46 (*n* = 1070); suicidal ideation severity at the height of the depression α = 0.86 (*n* = 1801), ICC = 0.72 (*n* = 1070); suicidal risk severity in the most recent week α = 0.80 (*n* = 221), ICC = 0.60 (*n* = 1070); suicidal risk severity at the height of the depression α = 0.88 (*n* = 221), ICC = 0.75 (*n* = 1070).

#### Comorbidity symptom scale

A previous study [29] showed that childhood emotional abuse, sexual abuse, somatic neglect, and total trauma score have significant effects on borderline personality disorder (BPD, *p* < 0.001, *p* = 0.038, *p* = 0.044, and *p* = 0.003, respectively), while emotional neglect and childhood trauma minimization have significant effects on dissociative disorders (*p* = 0.020 and *p* = 0.007, respectively). Also, BPD symptoms are often accompanied by a series of co-occurring phenomena, such as total self-esteem score, substance addiction, depression, and anxiety, but literature on the impact of this series of co-occurring mechanisms on TCE and suicidal behavior is scanty.

In the current study, the self-esteem level (SEL) developed by Rosenberg et al. was used to rate adolescents' overall feelings regarding self-worth and self-acceptance [30]. The scale consists of 10 entries, with 5 positive scorings and 5 negative scorings. The entries are scored on four levels: 1, very conforming; 2, conforming; 3, non-conforming; and 4, very non-conforming, with lower scores indicating a lower level of self-esteem. The Internet Addiction Impairment Index (IAII) [31] was adopted to evaluate the subjects' tendency and severity of Internet addiction. The scale has 20 items and is scored on a 5-point scale (1, rarely; 2, occasionally; 3, sometimes; 4, often; and 5, always), with higher scores indicating more serious Internet addiction. A score of ≥ 50 is considered the presence of Internet addiction. The internal consistency coefficient for the full scale was 0.94. Screen for Child Anxiety-Related Emotional Disorders (SCARED) [32] was chosen for self-assessment of anxiety. The scale has 41 items, each rated on three levels: 0, no symptoms; 1, partial anxiety; and 2, frequent anxiety. It covers five factors (generalized anxiety, somatization/panic, separation anxiety, social phobia, and school phobia), and a total score > 23 indicates the presence of anxiety. The Cronbach's alpha coefficient of the scale was 0.89.

### Quality control

To ensure the reliability and consistency of the questionnaire results, Wenwu Zhang served as the primary test. A pre-test was conducted before the study, where the purpose and data confidentiality were explained on-site. After the test, the questionnaires were collected by the lead physician and checked for double entries and missing answers. A total of 311 questionnaires were distributed and collected, with a 100% return rate. None of the questionnaires had missing data > 30% and logical errors.

### Data analysis method

After logical checks and proofreading, data entry was performed using Epidata 3.1 software. A multifactorial interactive analysis of TCE and suicidal ideation was performed using path analysis model construction. All variables in the model are described using the quantified values of the corresponding scales, that is, all variables are continuous variables. Following the recommendations of Hu and Bentler [33], we used several fit indices to evaluate model fit, including the Chi-square statistic (χ^2^), Chi-squared degree of freedom ratio (χ^2^/dƒ), the goodness-of-fit index (GFI), the comparative fit index (CFI), the root mean square error of approximation (RMSEA), the normed fit index (NFI), and non-normed fit index (NNFI). The alpha for the analysis was set to 0.05.

In addition, we adjusted the path of the model to meet the fitting conditions of the model with reference to the covariance coefficient between the variables given by the model. Finally, based on the established path model, we used the bootstrap estimation method [34] to test the mediating effect of self-esteem, Internet addiction, and anxiety on the impact of childhood traumatic experiences on suicidal ideation, and used the Sobel test to measure the significance of the mediation effect.

All analyses were conducted using the SPSS software, version 16.0.

## Results

Among the 311 subjects enrolled in the survey, 250 (80.39%) suffered traumatic experiences, such as emotional abuse, somatic abuse, sexual abuse, emotional neglect, or somatic neglect during childhood, 303 (97.43%) had suicidal ideation, 271 (87.14%) presented total SELs, 148 (47.59%) had varying degrees of Internet addiction, and 286 (91.96%) showed significant anxiety. The results showed that most of the subjects with NSSI behavior had traumatic experiences during childhood, and more individuals had suicidal ideation. Also, a large proportion of the subjects had comorbid symptoms of varying degrees and forms.

### Correlation analysis

The selection of factors in the path analysis model construction is crucial. DA Gould et al. [35] concluded, using cross-sectional sampling of abuse questionnaires, depression diagnostic inventories, and questions about suicide, that patients with a history of childhood abuse, especially sexual and emotional abuse, were at an increased risk of suicidal behavior. Joiner T E et al. [36], based on data from the American Comorbidity Survey, found that childhood physical and violent sexual abuse should be considered a greater risk factor for future suicide attempts and may cause a range of physical or psychological comorbidities. Based on these findings, factors such as TCE were considered outliers to evaluate the degree of influence on Suicidal ideation (SI). The variables SEL, Internet addiction (IA), and Anxious mood (AM) were used as internal derivatives to take over the relationship between external derivatives and SI (outliers mean variables in the model that are not affected by any other variables but affect others; internal derivatives are variables in the model that can be affected by any other variable). As shown in Table 1, the correlation analysis across the variables also supported the choice of model factors.

**Table 1:** Correlations across the main variables

	Average value	SD	SI	AM	SEL	IA	TCE
SI	2.482	0.438	1				
AM	48.399	16.245	0.452**	1			
SEL	21.685	3.803	-0.368**	-0.267**	1		
IA	49.084	21.025	0.199**	0.292**	-0.038	1	
TCE	54.383	14.833	0.365**	0.330**	-0.231**	0.120*	1

^*^ *p* < 0.05, **, *p* < 0.01

**Fig. 1:**
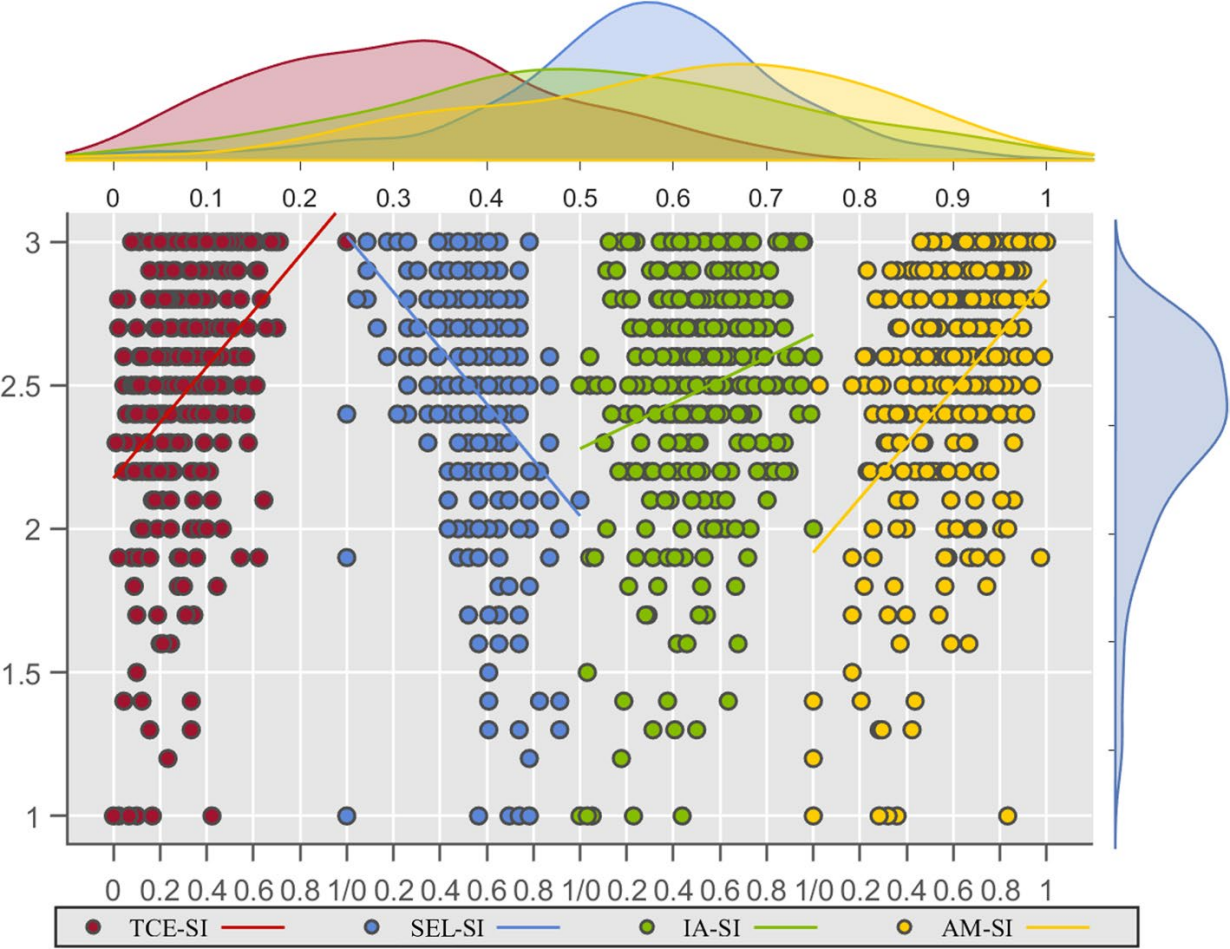
Scatter-trend of variables

The Pearson correlation coefficient was used to evaluate the strength of the correlations between suicidal ideation and anxiety, total self-esteem score, Internet addiction, and TCE. Figure 1 shows the trend of scattering distribution between SI and each of the other variables, and also the normalized probability density curve of each variable. As shown in Table 1, significant correlations were observed between SI and AM (*r* = 0.452, *p* < 0.01), SEL (*r* = -0.368, *p* < 0.01), IA (*r* = 0.199, *p* < 0.01), and TCE (*r* = 0.365, *p *< 0.01).

There was no significant correlation between SEL and IA (p > 0.05). Both the TCE (outer derivatives) and the AM, SEL, and IA (inner derivatives) showed significant correlations with each other, suggesting that there may be a complex intertwined effect between the variables.

### Path analysis model

**Fig. 2:**
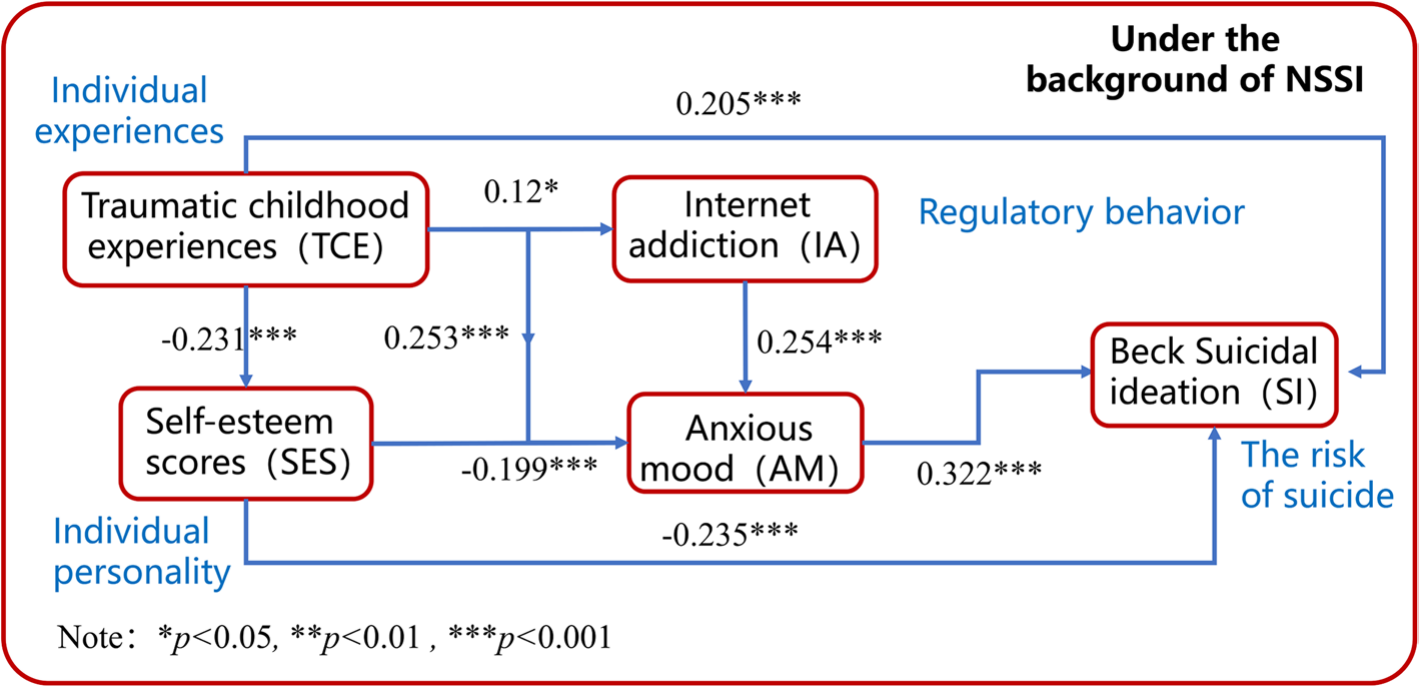
Path diagram of the path analysis model

The complex network of interactions across TCE, SI, low SEL, IA, and AM was further investigated using a path network analysis model, as shown in Fig. 2. Table 2 is the χ2, the GFI, the CFI, the RMSEA, and the weighted root mean square residual (WRMR) for model criterion. As shown in Table 2, the path analysis models constructed in the current work fit well.

**Table 2:** Model fitting index

Indicators	$\boldsymbol\chi^{\mathbf2}$	*p*	GFI	RMSEA	CFI	NFI	NNFI
Judgment standard	-	> 0.05	> 0.9	< 0.1	> 0.9	> 0.9	> 0.9
Model fitting value	2.562	0.278	0.996	0.030	0.997	0.988	0.986

The path relationships of the factors in the model have been plotted in Fig. 2. The standardized path coefficient values were -0.235 (z = -4.742, *p* < 0.01), 0.322 (z = 6.296, *p* < 0.01), and 0.205 (z = 4.047, *p* < 0.01) for the effects of SEL, AM, and TCE, respectively, on SI, indicating that self-esteem had a significant negative effect and anxiety and TCE had a significant positive effect on suicidal ideation. The standardized path coefficient values were -0.199 (z = -3.839, *p* < 0.01), 0.253 (z = 4.856, *p* < 0.01), and 0.254 (z = 4.998, *p* < 0.01) for SEL, TCE, and IA, respectively, on AM, indicating that self-esteem had a significant negative effect and TCE and Internet addiction had a significant positive effect on anxiety. The standardized path coefficient values were -0.231 (z = -4.193, *p* < 0.01), and 0.120 (z = 2.130, *p* < 0.05) for the effects of TCE on SEL and IA, respectively, indicating that childhood traumatic experiences have a significant negative effect on SELs and a significant positive effect on Internet addiction.

Moreover, from the structural equation model shown in Fig. 2, it can be seen that in addition to directly affecting SI, TCE also acts on SI through four different indirect paths. The results of the Bootstrap sampling test of the indirect effects are shown in Table 3. For the four intermediary paths ' TCE → SEL → SI', ' TCE → AM → SI', ' TCE → SEL → AM → SI', ' TCE → IA → AM → SI', the 95% intervals are 0.021 ~ 0.096, 0.033 ~ 0.129, 0.004 ~ 0.029, < 0.001 ~ 0.023, all of which do not include the number 0, so it shows that the above-mentioned mediating effect path exists.

**Table 3:** Summary of indirect effect analysis

Path	Effect	Boot SE	BootLLCI	BootULCI	z
TCE → SEL → SI	0.002	0.019	0.021	0.096	0.086
TCE → AM → SI	0.002	0.024	0.033	0.129	0.091
TCE → SEL → AM → SI	< 0.001	0.006	0.004	0.029	0.064
TCE → IA → AM → SI	< 0.001	0.006	< 0.001	0.023	0.045

BootLLCI refers to the lower limit of the 95% interval of Bootstrap sampling, and BootULCI refers to the upper limit of the 95% interval of Bootstrap sampling

### Mediating effect

**Fig. 3:**
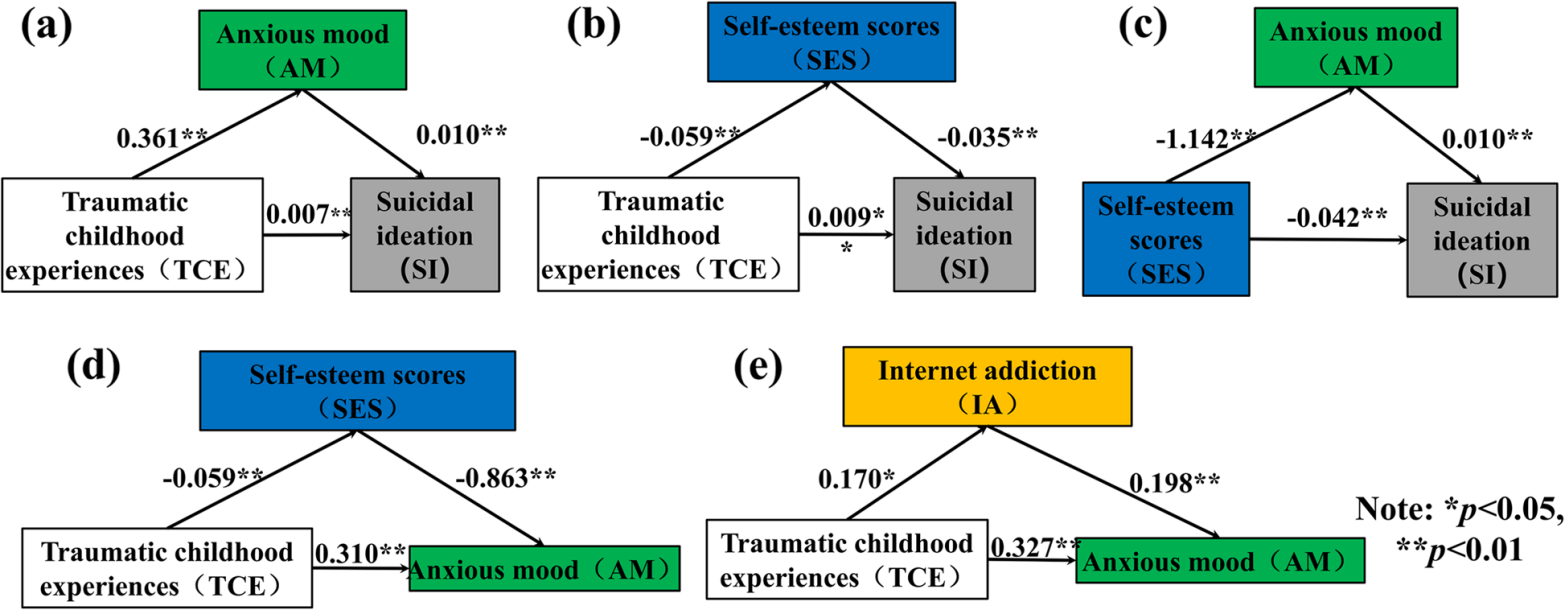
The mediating model

In the analytical work (Fig. 2), TCE play a mediating role in influencing suicidal ideation, and psychological and even physical comorbidities, such as SELs, Internet addiction, and anxiety, may indirectly influence the individual's suicidal ideation. To this end, this work further conducted an independent mediation test analysis on the variables involved in the mediation pathway of the above structural equation model to explore the mediation effect of related symptoms in adolescent patients diagnosed with NSSI. Figure 3 shows the schematic diagram of the mediation model of a range of comorbidities between TCE and suicidal ideation.

As shown in Fig. 3a, b, the mediation models constructed with anxiety/SELs as mediating variables, childhood traumatic experiences as independent variables, and suicidal ideation as dependent variables were significant for the study subjects, and the indirect effects of childhood traumatic experiences were B = 0.004/0.002, SE = 0.029/0.022, 95% CI [0.071, 0.181]/[0.027, 0.115]. In addition, the regression coefficient for the direct association between TCE and suicidal ideation was significant (B = 0.011, 95% CI [0.008, 0.014]), before the inclusion of anxiety/ SEL as a mediating variable. The correlation between childhood traumatic experiences and suicidal ideation remained significant when anxiety/ SELs were added as mediating variables in the analysis (B = 0.007/0.009, 95% CI [0.004, 0.010]/[0.006, 0.012]). The effect share of anxiety/ SELs in the total effect of TCE on suicidal ideation was Ef = 33.574%/18.968% (calculated using Eq. (1)), which indicates that anxiety/ SEL partially mediates the relationship between TCE and suicidal ideation.

1 $${E}_{f}={a}^{*}b/c$$

As shown in Fig. 3d, e, the mediation models constructed with SEL/internet addiction as mediating variables, childhood traumatic experiences as independent variables, and anxiety as dependent variables were significant for the study subjects, and the indirect effects of childhood traumatic experiences were B = 0.051/0.034, SE = 0.020/0.017, 95% CI [0.013,0.092]/[0.002, 0.066]. In addition, the regression coefficient for the direct association between TCE and anxiety was significant (B = 0.361, 95% CI [0.246, 0.476]), before the inclusion of SEL/internet addiction as a mediating variable. The correlation between childhood traumatic experiences and anxiety remained significant when SEL/internet addiction was added as a mediating variable in the analysis (B = 0.310/0.327, 95% CI [0.194, 0.426]/[0.215, 0.439]). The effect share of SEL/internet addiction in the total effect of TCE on anxiety was Ef = 14.152%/9.298%, suggesting that SEL/internet addiction partially mediates the relationship between TCE and anxiety.

Table 4 summarizes the potential mediating effects included in the model. c denotes the regression coefficient when the independent variable *X* is on the dependent variable *Y* (when there is no mediating variable *M* in the model), i.e., the total effect. a denotes the regression coefficient when *X* is on *M*, *b* denotes the regression coefficient when *M* is on* Y*, and a*b is the product of *a* and *b*, i.e., the mediating effect. *c'* denotes the regression coefficient when *X* is on *Y* (when the model As shown in Table 3, with the introduction of the mediating variable *M* (i.e., anxiety/ SEL/Internet addiction), the direct effects (i.e., regression coefficients) of childhood traumatic experiences on suicidal ideation/anxiety were significant. The direct effects (i.e., regression coefficients) of childhood traumatic experiences on suicidal ideation/anxiety showed a decreasing trend (TCE → AM → SI: 0.011 decreased to 0.007; TCE → SEL → SI: 0.011 decreased to 0.009; SRE → AM → SI: -0.042 decreased to -0.031; TCE → SEL → AM: 0.361 decreased to 0.310; TCE → IA → AM: 0.361 decreased to 0.327), indicating the direct effect of childhood traumatic experiences on suicidal ideation/anxiety. This suggested that the complex association between childhood traumatic experiences and suicidal ideation/anxiety is further influenced by various mediating variables. The 95% confidence intervals calculated using the Bootstrap sampling method did not cover 0, which verified that the model constructed had significant mediating effects.

**Table 4:** Summary of intermediary role test results

	*c* total effect	a	b	a*b Intermediary Effect	c’ Direct effect	a*b (95% Boot Cl)	Effectiveness Ratio (a*b/c)
TCE → AM → SI	0.011 **	0.361 **	0.010 **	0.004	0.007 **	0.071–0.181	33.574%
TCE → SEL → SI	0.011 **	-0.059 **	-0.035 **	0.002	0.009 **	0.027–0.115	18.968%
SEL → AM → SI	-0.042 **	-1.142 **	-0.010 **	-0.012	-0.031 **	-0.159–0.050	27.633%
TCE → SEL → AM	0.361 **	-0.059 **	-0.863 **	0.051	0.310 **	0.013–0.092	14.152%
TCE → IA → AM	0.361 **	0.170 **	0.198 **	0.034	0.327 **	0.002–0.066	9.298%

^**^ *p* < 0.01

## Discussion

In the current study, adolescents who were diagnosed with NSSI for the first time were included, and after correlational analysis of the influencing factors, we constructed a structural equation model to further assess the complex interactions across TCE, suicidal ideation, low self-esteem, Internet addiction, and anxiety. It was found that up to 80.39% of the subjects with NSSI behavior experienced at least one type of TCE, and this proportion was higher in individuals with SI. In addition, a large proportion of the subjects had varying degrees and forms of comorbid symptoms that also directly or indirectly mediated the development of IA.

The proportion of the study subjects with TCE was much higher than the prevalence of TCE in a community sample of adolescents [37], which is consistent with the results of a systematic review by Liu R T et al. [38]. As a prevalent global health problem, child maltreatment is closely associated with and increases the risk of negative mental health outcomes later in life [39]. Children have immature psychological development, poor personality stability, and incomplete cognition if they experience traumatic events, such as physical, psychological, and emotional abuse during childhood. They tend to repeatedly review their emotional experiences and emotional states, and this negative cognition causes a snowballing accumulation of emotions. Such complex negative emotions add to the psychological burden of patients and make them more prone to psychological problems [40]. The quality-stress theory of suicide proposed by John [41] suggested that childhood traumatic experiences are long-term stressful events that have a great negative impact on the individual's personality, cognition, emotion, coping style, and other psychological processes, causing the individual to have more negative psychological experiences and reduce their coping ability when they encounter setbacks during growth, which ultimately increases the risk of suicide. In addition, TCE may pose a long-term risk for non-suicidal self-harm that may carry over into adulthood.

Although the close relationship between TCE, NSSI, and suicide has been demonstrated in previous studies [42, 43], further exploration of the moderators and mediating pathways linking TCE to SI would be valuable in advancing risk stratification strategies and identifying promising targeted interventions. However, many studies [44, 45] have shown that suicidal ideation generated under non-suicidal self-injury behaviors is highly related to recent events of patients, which is often accompanied by a strong "nearest time principle". Cerutti R et al. [46] believe that between the NSSI behaviors reflected in early adolescents and suicidal ideation, there are multiple stressors in school, family, and society in the current adolescent stage, and the disappearance and transfer of the current stressors often lead to NSSI as time goes by. Regression of behavior and suicidal ideation. In a study [47] on the relationship between employment stress, self-esteem and suicidal ideation among college students, it was also shown that those who had recently experienced negative life stress had lower self-esteem than those who had experienced negative life events in the past six months to a year. More likely to present with higher levels of suicidal ideation. Vito S. Guerra et al. [48] observed in a sample of American veterans: the post-traumatic stress disorder and suicidal ideation reflected by the sample mainly came from the previous combat experience; the study found that with the integration of life out of the army and ordinary life, suicide Ideation and its comorbid symptoms, such as depression, showed a gradually decreasing level. Therefore, NSSI and its suicidal ideation are highly correlated with patients' "here and now" stressors and show a decreasing trend over time. Current evidence on the mediating effects of TCE and suicidal ideation/anxiety is mostly limited to a single risk factor or outcome, and few studies have examined the moderating role of multiple factors between TCE and suicidal ideation, SELs, and anxiety in individuals with NSSI behaviors. According to Beck's cognitive susceptibility model [49], individuals' early negative experiences lead to negative cognition of themselves and the surrounding environment, which in turn lowers adolescents' SELs. However, adolescents with low self-esteem have low self-identity and self-acceptance ability, which makes them lack the ability to control life and respond positively when they encounter setbacks, and ultimately increases the risk of suicide [50], suggesting that SEL as an individual characteristic plays an important mediating effect between TCE and suicide. Childhood traumatic experiences can cause the development of negative perceptions of the patients themselves as unworthy of love and others as unreliable and untrustworthy. They tend to maintain the patients in a long-term psychological state of worry, fear, anxiety, and trepidation, and these negative cognitive patterns are major risk factors for anxiety and depressive symptoms when individuals grow up. Brown et al.'s recent path analysis model also demonstrated partial mediating effects of emotional abuse and complete mediating effects of sexual abuse, depression, and anxiety on NSSI [16]. Depression/anxiety-self-esteem theory [49, 51, 52] suggested that low self-esteem is one of the most important susceptibility factors for depression/anxiety, suggesting that adolescents with low self-esteem are more likely to develop anxiety or even depression after negative life events, which suggests that we mediate the transmission relationship among factors. Furthermore, based on some previous studies reporting childhood trauma and its subtypes, such as emotional, physical, and sexual abuse, as factors associated with Internet addiction or online gaming disorders in different populations [53–57], using the Internet may be a more popular coping strategy to avoid experiences focused on trauma or bullying and stressful life events or anxiety [58], and for this reason, we also took the factor of Internet addiction included in the consideration of the mediated model.

The path analysis model constructed in the current study further validates the above inferences. However, only a few relevant studies supporting the above findings are available and most of them focused on adolescents older than 16 years old. A multitopic-multipath model was constructed in the current study, and we found significant direct as well as indirect moderating effects of TCE on IA/anxiety. Specifically, individuals with TCE were more likely to exhibit low levels of SEL as well as IA behaviors, which in turn could be directly associated with suicidal ideation. In addition, TCE and IA behaviors induce anxiety, and anxiety and TCE can directly trigger suicidal ideation. Overall, childhood abuse and suicidality in the context of NSSI have complex intertwined influential relationships that are often reflected in mediating effects of individual personality problems (low self-esteem, anxiety, internet addiction, etc.), which is supported by the pathway model shown in Fig. 2, while the mediating relationships shown in Fig. 3 suggest a multiple mediating effect among the variables in the pathway network. Unlike the traditional chain mediation model, the factor of Internet addiction is not directly related to SI, so we obtained a special chain mediation model with a partial moderating effect. This result further enriched TCE's discussion on the NSSI mechanism, validated and extended the relevant literature, and showed certain theoretical implications.

For clinical application, the results of this study suggest that it is necessary to conduct early screening and intervention of TCE in the crisis intervention of suicide, to prevent and treat NSSI, anxiety, depression, and other related psychological problems at an early stage. The focus should also be on diagnosing and assessing individuals' anxiety status and Internet addiction and developing targeted interventions for individuals with corresponding risk factors. Parents and schools should also focus on the cultivation of self-esteem thoughts in adolescents.

### Limitations

There are also certain shortcomings to the current study. Firstly, the study was cross-sectional. Although previous studies and theories have provided a solid foundation for this study, it is still difficult to draw an exact causal relationship. There is a need for a longitudinal study to further explore the causal relationships among variables. Secondly, our study population was NSSI patients, and the general population was not included as a control to account for confounding factors. At the same time, this study includes two additional limitations: (1) the possibility of recalling bias, and (2) the limitations of generalizability as the effect of psychological trauma are often influenced by cultural and social factors.

## Conclusion

In conclusion, we constructed a multitopic-multipath model to assess the relationship between distal and proximal factors for the presence of suicidality in individuals with TCE and NSSI behaviors and highlighted the possibility that childhood familial factors may contribute to psychiatric comorbid symptoms and suicidal behavior. Specifically, our findings suggest that mental health professionals need to assess suicide attempts in individuals with NSSI and explore both childhood abuse history, personality traits, emotional status (e.g., anxiety and depression), and substance addiction. Interventions that recognize the multifactorial nature of TCE and SI and include efforts to improve self-esteem and strategies to regulate emotions may prevent the subsequent development and maintenance of suicidal ideation in patients with TCE and NSSI. Our data provide a theoretical basis for identifying individuals with appropriate risk factors for targeted interventions.

No potential conflict of interest was reported by the authors.

## Data Availability

The datasets used and/or analysed during the current study available from the corresponding author on reasonable request.
